# Marital status impact on the outcomes of patients admitted for acute decompensation of heart failure: A retrospective, single‐center, analysis

**DOI:** 10.1002/clc.24053

**Published:** 2023-06-12

**Authors:** Gil Marcus, Natalia Kofman, Shiri L. Maymon, Elad Asher, Dan Loberman, David Pereg, Shmuel Fuchs, Sa'ar Minha

**Affiliations:** ^1^ Cardiology, Shamir Medical Center Be'er‐Yaakov Israel; ^2^ Sackler School of Medicine Tel‐Aviv University Ramat‐Aviv Israel; ^3^ The Jesselson Integrated Heart Center, Shaare Zedek Medical Center, Faculty of Medicine Hebrew University Jerusalem Israel; ^4^ Cape Cod Hospital, Brigham and Women's Hospital, Harvard Medical School Division of Cardiac Surgery Boston Massachusetts USA; ^5^ Cardiology Department Meir Medical Center Kfar‐Saba Israel

**Keywords:** acute decompensated heart failure, clinical outcomes, marital status

## Abstract

**Background:**

Conflicting evidence exists regarding the association between marital status and outcomes in patients with heart failure (HF). Further, it is not clear whether type of unmarried status (never married, divorced, or widowed) disparities exist in this context.

**Hypothesis:**

We hypothesized that marital status will be associated with better outcomes in patients with HF.

**Methods:**

This single‐center retrospective study utilized a cohort of 7457 patients admitted with acute decompensated HF (ADHF) between 2007 and 2017. We compared baseline characteristics, clinical indices, and outcomes of these patients grouped by their marital status. Cox regression analysis was used to explore the independency of the association between marital status and long‐term outcomes.

**Results:**

Married patients accounted for 52% of the population while 37%, 9%, and 2% were widowed, divorced, and never married, respectively. Unmarried patients were older (79.8 ± 11.5 vs. 74.8 ± 11.1 years; *p* < 0.001), more frequently women (71.4% vs. 33.2%; *p* < 0.001), and less likely to have traditional cardiovascular comorbidities. Compared with married patients, all‐cause mortality incidence was higher in unmarried patients at 30 days (14.7% vs. 11.1%, *p* < 0.001), 1 year, and 5 years (72.9% vs. 68.4%, *p* < 0.001). Nonadjusted Kaplan‐Meier estimates for 5‐year all‐cause mortality by sex, demonstrated the best prognosis for married women, and by marital status in unmarried patients, the best prognosis was demonstrated in divorced patients while the worst was recorded in widowed patients. After adjustment for covariates, marital status was not found to be independently associated with ADHF outcomes.

**Conclusions:**

Marital status is not independently associated with outcomes of patients admitted for ADHF. Efforts for outcomes improvement should focus on other, more traditional risk factors.

## Graphical abstract

- As opposed to the unadjusted model, a multivariate cox regression model demonstrated marital status not to be an independent index associated with 5‐year mortality (HR 0.98 [0.91–1.05], *p* = 0.6).

- Efforts for improving the long‐term outcomes of patients with heart‐failure should focus on concomitant medical conditions (e.g., anemia and COPD).

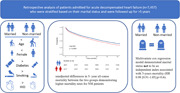

## 1 INTRODUCTION

Heart failure (HF) is a leading cause of morbidity and mortality worldwide. It is estimated that >6 million adult Americans were diagnosed with HF between 2015 and 2018, and similar trends are seen in Europe.^1^, ^2^ Socioeconomic status (SES) impacts both the incidence and the outcome of HF.^3^, ^4^ Since socioeconomic status is multifactorial, the exact impact of different determinants of SES on HF is less clear. Marriage is one of the closest and most intimate social support environments, nevertheless, conflicting evidence exists regarding the association between marital status and outcomes in patients with HF.^5^, ^6^, ^7^, ^8^, ^9^ We thus explored the impact of marital status on both the short and long‐term outcomes of patients admitted with acute decompensated HF (ADHF).

## 2 METHODS

This single‐center, observational retrospective cohort study utilized data from all adult patients admitted to Shamir Medical Center with ADHF between January 1, 2007, and December 31, 2017. The last date for all‐cause mortality was December 31, 2020. Eligible patients were those older than 18 years admitted to an internal medicine department (IMD), who were clinically diagnosed with ADHF upon admission (ICD‐9 codes: 428.xx, 429.xx, and 514) and subsequently discharged with a similar diagnosis. We elected to include only HF patients admitted to IMD, and not patients admitted to cardiology departments, as we showed in a previous study that these two populations are distinctively different from each other, with patients admitted to IMD being more reflective of the general population of HF patients.^10^ This study was approved by the local institutional review board at Shamir Medical Center and patient consent was waived because of the retrospective nature of the data and analysis.

Demographic, clinical, laboratory, and follow‐up data including readmission within 30 days were extracted from the hospital's electronic medical record, and all‐cause mortality from Israel's ministry of interior affairs database. As described earlier,^10^ a separate data set was created to consolidate medical therapy by drug groups both on admission and at discharge.

For the present analysis, patients were grouped based on self‐reported marital status as either “married” (including married or attached) or “unmarried” (including single (i.e., never married), divorced, and widowed). A comparison between married and unmarried patients for demographic, clinical, and outcomes indices was performed. Categorical variables are expressed as numbers and percentages, and continuous variables are expressed as either mean ± standard deviation (SD) or median and inter‐quartile range according to the normality of distribution. Fisher's exact test was used to compare categorical variables. The two‐paired student *t* test was used to compare normally distributed continuous variables, and the Mann–Whitney *U* test was used to compare non‐normally distributed continuous variables. Normality was tested by the Kolmogorov–Smirnov test.

Logistic regression analysis was used to explore if marital status was independently associated with 30‐day readmission and 30‐day all‐cause mortality. Cox logistic regression analysis was further utilized to explore indices associated with 5‐year all‐cause mortality. Co‐variates used in all models were forced based on their relevance to our research question (marital status, sex) or their known association with outcomes of HF patients (age,^11^ diabetes, ischemic heart disease,^12^ chronic kidney disease,^13^ atrial fibrillation,^14^ chronic obstructive pulmonary disease,^15^ anemia,^16^ and peripheral vascular disease^17^).

All statistical tests were two‐sided. *p* < 0.05 was considered significant.

Statistical analysis was carried out using R: A Language and Envirounmarriedent for Statistical Computing, R Core Team, R Foundation for Statistical Computing, Vienna, Austria, 2020, https://www.R-project.org.

## 3 RESULTS

A total of 7457 patients admitted with a diagnosis of ADHF between 2007 and 2017 with reported marital status, were included in the analysis. Of those, 3904 (52%) patients were married. In the unmarried group, most patients (2722;77.1%) were widowed, 636 (18%) were divorced and 173 (4.9%) were never married.

As detailed in Table 1, compared with patients in the married population, unmarried patients were older (79.8 ± 11.5 vs. 74.8 ± 11.1 years; *p* < 0.001), and had a higher prevalence of females (71.4% vs. 33.2%; *p* < 0.001). Further, compared with married patients, unmarried patients were less likely to have traditional cardiovascular comorbidities such as diabetes mellitus, smoking, peripheral vascular disease, chronic kidney disease, and ischemic heart disease and were less likely to be chronically prescribed with angiotensin receptor binders, mineralocorticoid receptor antagonist, statins and antithrombotic medications. Patients in the unmarried group had a higher prevalence of normal left‐ventricular function compared with married patients.

**Table 1:** Baseline demographic, pharmacological, clinical, and laboratory indices of patients admitted with acute decompensated heart failure.

Characteristics	Married (*n* = 3904)	Unmarried (*n* = 3553)	*p* Value
Age, mean ± SD, years	74.9 ± 11.1	79.8 ± 11.5	<0.001
Female sex, *n* (%)	1297 (33)	2538 (72)	<0.001
Heart failure as primary diagnosis, *n* (%)	1781 (45.6)	1552 (43.7)	0.10
Past medical history			
Hypertension, *n* (%)	678 (17.4)	629 (17.7)	0.72
Diabetes mellitus, *n* (%)	2130 (54.6)	1620 (45.6)	<0.001
Obesity, *n* (%)	877 (22.5)	734 (20.7)	0.06
Smoking, *n* (%)	707 (18.1)	373 (10.5)	<0.001
Ischemic heart disease, *n* (%)	1725 (44.2)	1299 (36.6)	<0.001
COPD, *n* (%)	652 (16.7)	582 (16.4)	0.73
PVD, *n* (%)	338 (8.7)	205 (5.8)	<0.001
Chronic kidney disease, *n* (%)	1416 (36.3)	1116 (31.4)	<0.001
Atrial fibrillation, *n* (%)	1197 (30.7)	1210 (34.1)	0.002
Chronic medical therapy			
Alpha blockers, *n* (%)	434 (11.1)	391 (11.0)	0.91
Beta blockers, *n* (%)	1394 (35.7)	1243 (35.0)	0.53
Calcium channel blockers, *n* (%)	954 (24.4)	986 (27.8)	0.001
ACE inhibitors, *n* (%)	720 (18.4)	668 (18.8)	0.71
ARB, *n* (%)	469 (12.0)	345 (9.7)	0.002
Aldactone, *n* (%)	119 (3.0)	69 (1.9)	0.003
Antiarrhythmic, *n* (%)	265 (6.8)	233 (6.6)	0.73
Antiplatelets, *n* (%)	1685 (43.2)	1419 (39.9)	0.005
Oral anticoagulant, *n* (%)	566 (14.5)	515 (14.5)	1
Statin, *n* (%)	1602 (41.0)	1258 (35.4)	<0.001
Other lipid lowering, *n* (%)	101 (2.6)	42 (1.2)	<0.001
Digoxin, *n* (%)	210 (5.4)	183 (5.2)	0.70
Diuretic, *n* (%)	2180 (55.8)	2002 (56.3)	0.68
Combination pills, *n* (%)	55 (1.4)	50 (1.4)	1
Laboratory indices			
Hemoglobin, g/dL [IQR]	11.70 [10.30–13.10]	11.50 [10.30–12.80]	0.001
WBC, K/µL [IQR]	8.90 [7.00–11.70]	9.30 [7.20−12.40]	<0.001
Urea mg/dL [IQR]	51.70 [36.90–78.20]	50.85 [36.80–77.47]	0.78
Sodium, mmol/L [IQR]	138.00 [135.00–141.00]	138.00 [134.00–141.00]	0.003
Creatinine, mg/dL [IQR]	1.14 [0.87–1.63]	1.07 [0.83–1.51]	<0.001
Echocardiography			
Left ventricular function			<0.001
Normal (>50%), *n* (%)	562 (48)	537 (57)	
Mild dysfunction (40%–50%), *n* (%)	193 (16)	119 (12)	
Moderate dysfunction (30%–40%), *n* (%)	297 (25)	198 (21)	
Severe dysfunction (<30%), *n* (%)	128 (11)	95 (10)	
Any valvular disease, *n* (%)	532 (13.6)	507 (14.3)	0.4
Mitral valve disease, *n* (%)	331 (8.5)	277 (7.8)	0.30
Aortic valve disease, *n* (%)	282 (7.2)	293 (8.2)	0.11
Tricuspid valve disease, *n* (%)	86 (2.2)	84 (2.4)	0.70

Abbreviations: ACEi, angiotensin‐converting enzyme inhibitor; ARB, angiotensin II receptor blocker; COPD, chronic obstructive pulmonary disease; EF, ejection fraction; IQR, interquartile range; PVD, peripheral vascular disease; SD, standard deviation; WBC, white blood cell count.

Procedures performed during admission and medication prescribed at discharge are detailed in Table 2. Save for percutaneous coronary intervention which was performed with higher prevalence in the married patients group (5.1% vs. 3.4%, *p* < 0.001), no differences were noted in the prevalence of procedures performed during admission. Beyond the differences in medical therapy prescribed on admission, although the rate of diuretic and beta‐receptor blocker prescription at discharge was high, compared with married patients, unmarried patients were less likely to be prescribed these drugs.

**Table 2:** In‐hospital procedures and discharge prescriptions of patients admitted with acute decompensated heart failure.

	Married (*n* = 3904)	Unmarried (*n* = 3553)	*p* Value
Procedures performed during hospitalization			
Cardiac stress test, *n* (%)	6 (0.2)	2 (0.1)	0.35
Cardiac nuclear study, *n* (%)	0 (0.0)	1 (0.0)	0.96
Diagnostic coronary angiography, *n* (%)	103 (2.6)	70 (2.0)	0.07
Percutaneous coronary intervention, *n* (%)	199 (5.1)	121 (3.4)	<0.001
CABG, *n* (%)	38 (1.0)	24 (0.7)	0.20
Permanent pacemaker implanted, *n* (%)	36 (0.9)	30 (0.8)	0.48
Cardiac resynchronization therapy, *n* (%)	9 (0.2)	2 (0.1)	0.10
Dialysis, *n* (%)	63 (1.6)	43 (1.2)	0.17
Discharge medications			
Alpha blockers, *n* (%)	605 (15.5)	535 (15.1)	0.62
Beta blockers, *n* (%)	2,169 (55.6)	1828 (51.4)	<0.001
Calcium channel blockers, *n* (%)	1,269 (32.5)	1225 (34.5)	0.08
ACEi, *n* (%)	1,043 (26.7)	933 (26.3)	0.67
ARBs, *n* (%)	603 (15.4)	467 (13.1)	0.005
Aldactone, *n* (%)	132 (3.4)	75 (2.1)	0.001
Antiarrhythmic, *n* (%)	349 (8.9)	302 (8.5)	0.53
Antiplatelets, *n* (%)	2,338 (59.9)	1978 (55.7)	<0.001
Oral anticoagulant, *n* (%)	1,041 (26.7)	951 (26.8)	0.94
Statin, *n* (%)	2,281 (58.4)	1757 (49.5)	<0.001
Other lipid lowering, *n* (%)	124 (3.2)	54 (1.5)	<0.001
Digoxin, *n* (%)	179 (4.6)	158 (4.4)	0.82
Diuretic, *n* (%)	3,023 (77.4)	2679 (75.4)	0.04
Combination pills, *n* (%)	137 (3.5)	91 (2.6)	0.02
Other heart failure medications, *n* (%)	2 (0.1)	0 (0.0)	0.52

Abbreviations: ACEi, angiotensin‐converting enzyme inhibitor; ARB, angiotensin II receptor blocker; CABG, coronary artery bypass graft.

**Figure 1:**
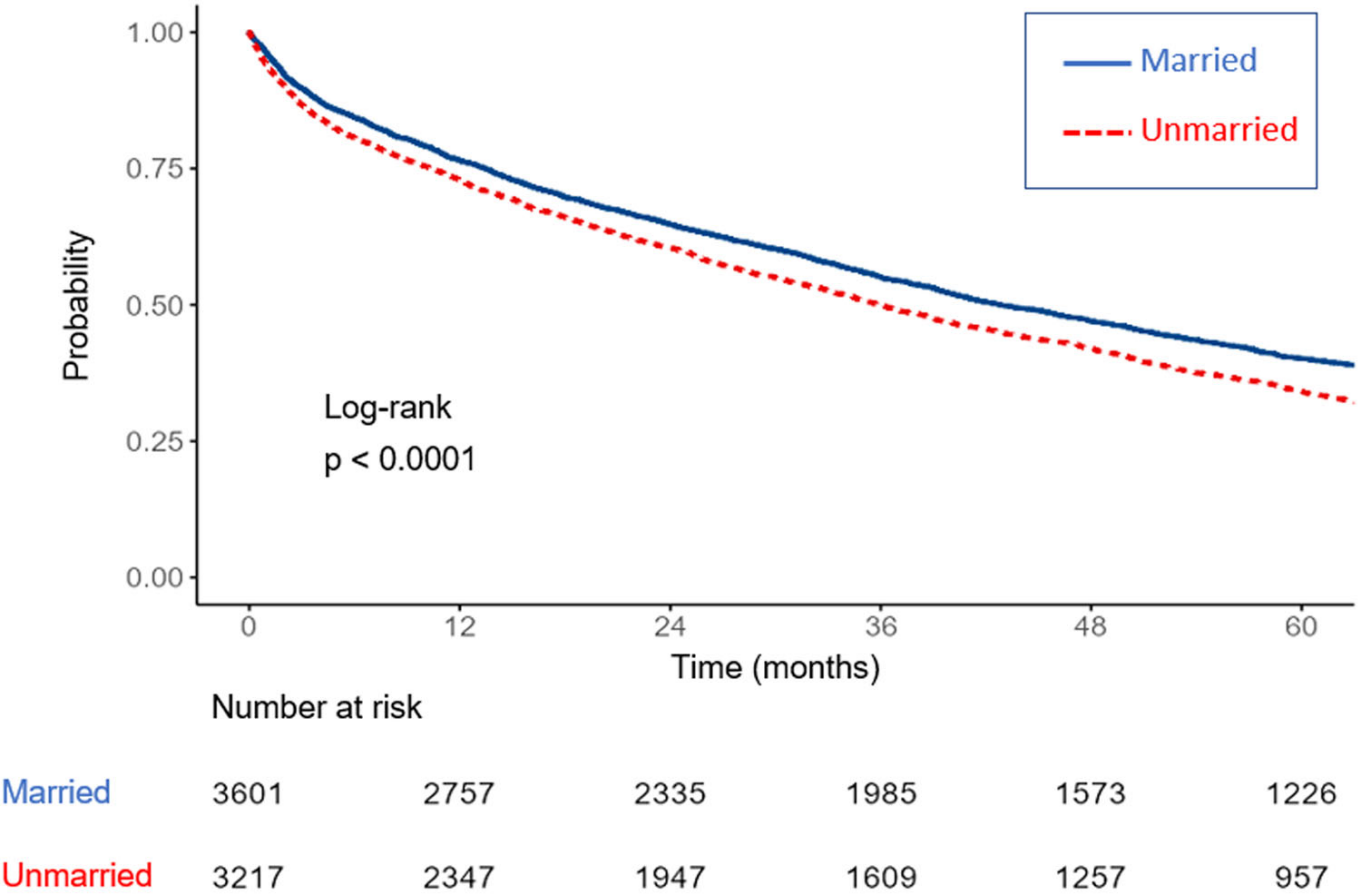
Kaplan–Meier survival estimate for patients admitted for acute decompensated heart failure stratified by marital status.

**Figure 2:**
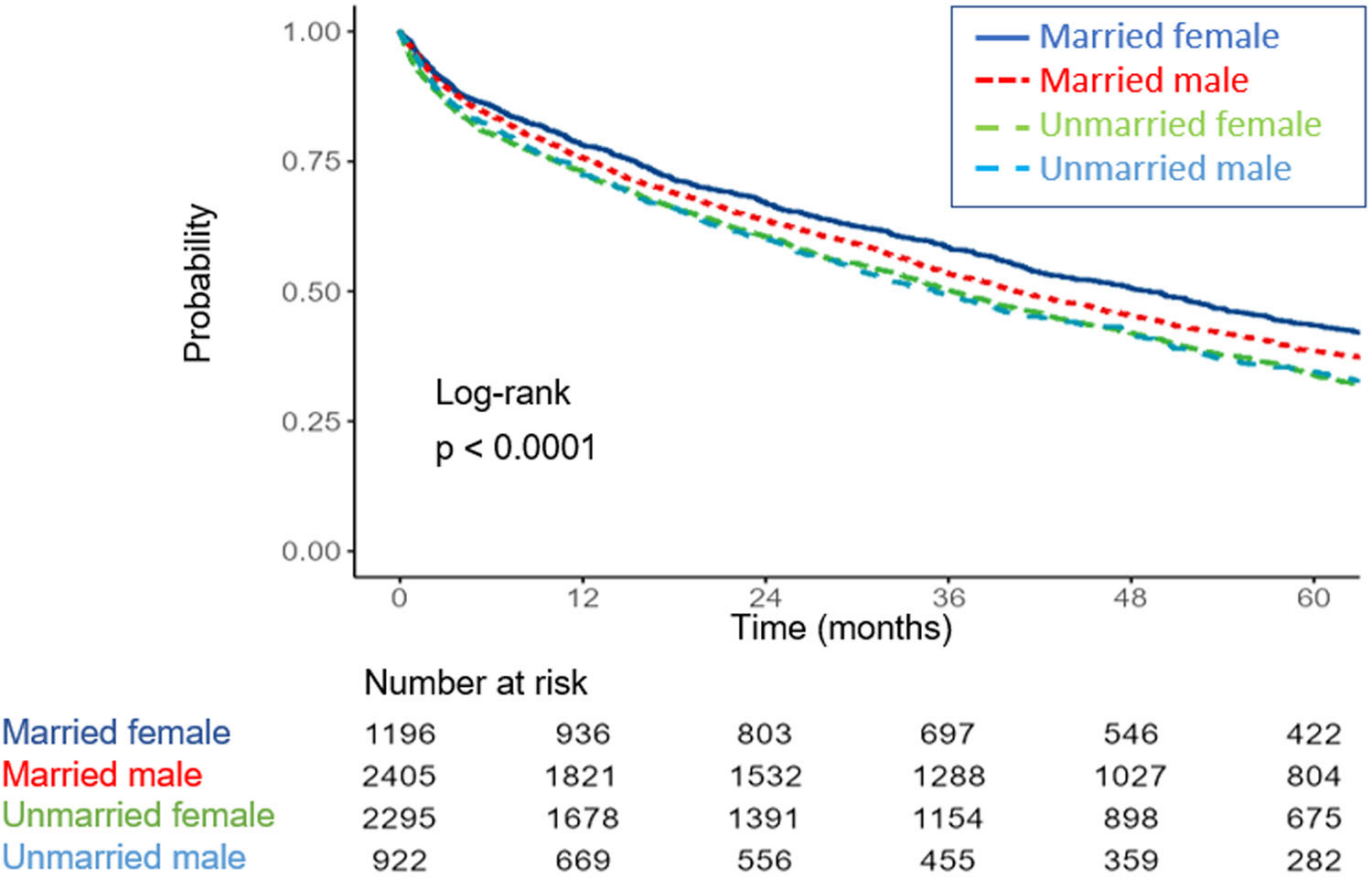
Kaplan–Meier survival estimate for patients admitted for acute decompensated heart failure stratified by marital status and gender.

**Figure 3:**
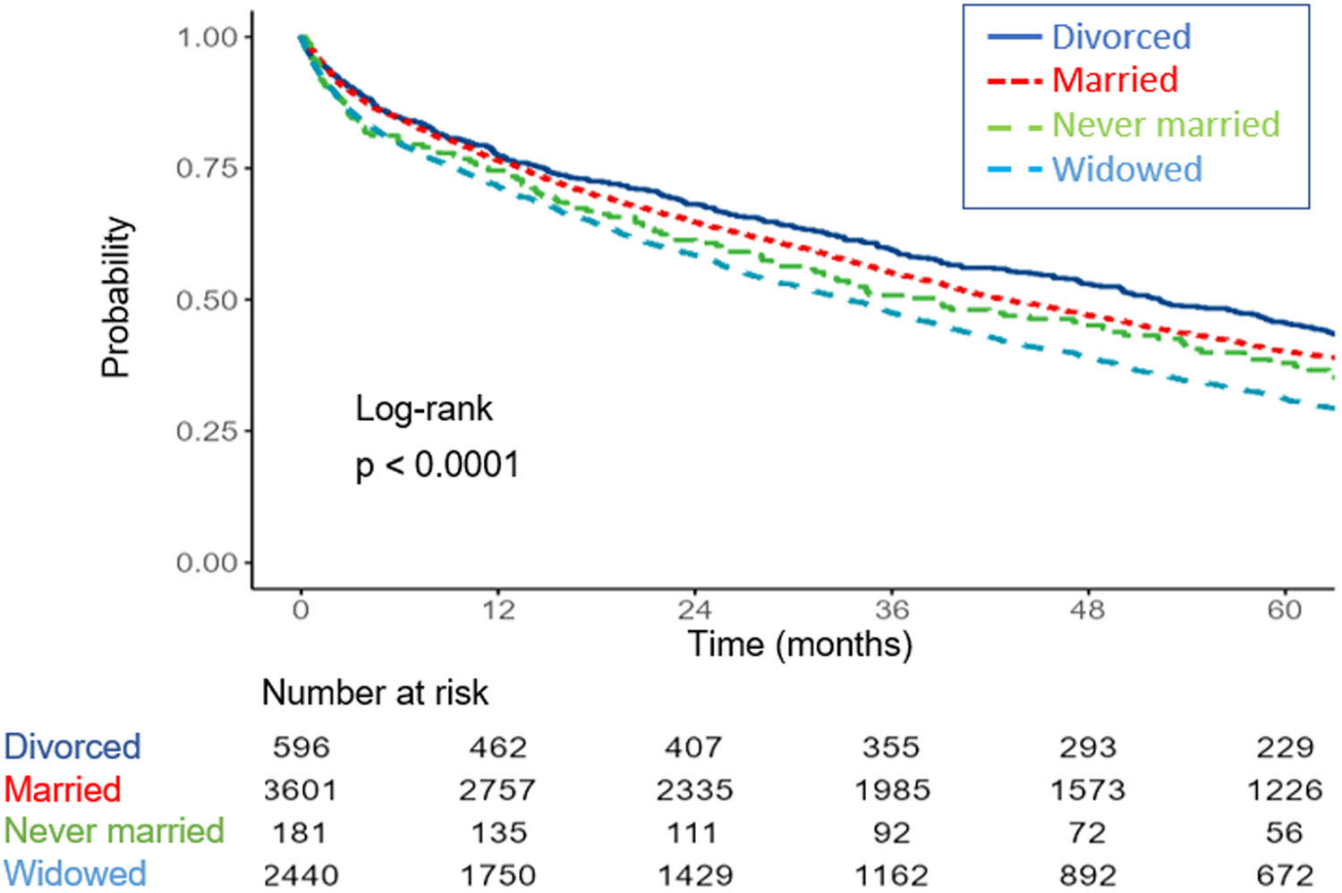
Kaplan–Meier survival estimate for patients admitted for acute decompensated heart failure stratified by four optional marital status.

The outcomes of the two study groups are detailed in Table 3. The incidence of 30‐day readmission did not differ between the groups (22.2% married vs. 20.4% unmarried; *p* = 0.08). On the other hand, all other explored outcome indices, including length of stay, all‐cause mortality at 30 days, and long‐term all‐cause mortality rates were higher in the unmarried group. Figure 1 depicts the unadjusted differences in 5‐ year all‐cause mortality between the two groups demonstrating higher mortality rates for unmarried patients. As depicted in Figure 2, stratifying outcomes by gender and marital status, married women had the highest survival rates at 5 years, followed by married men and unmarried patients (log rank < 0.001). Figure 3 depicts the unadjusted survival estimate of patients stratified into four marital status options. The highest survival rate was demonstrated in divorced patients while widowed patients had the worse prognosis at 5 years (log rank < 0.001).

**Table 3:** Clinical outcomes of patients admitted with acute decompensated heart failure.

Outcomes	Married (*n* = 3904)	Unmarried (*n* = 3553)	*p* Value
Length of stay, days [IQR]	5 [3–10]	6 [3–10]	0.007
In hospital mortality, *n* (%)	298 (7.6)	330 (9.3)	0.011
Readmission within 30 days, *n* (%)	801 (22.2)	659 (20.4)	0.08
Mortality within 30 days, *n* (%)	434 (11.1)	522 (14.7)	<0.001
Readmission or mortality within 30 days, *n* (%)	1163 (29.8)	1090 (30.7)	0.41
All‐cause mortality at 1 year, *n* (%)	1134 (29.0)	1193 (33.6)	<0.001
All‐cause mortality at 3 years, *n* (%)	1,908 (48.9)	1936 (54.5)	<0.001
All‐cause mortality at 5 years, *n* (%)	2671 (68.4)	2590 (72.9)	<0.001

Abbreviation: IQR, interquartile range.

Multivariate analyses for indices independently associated with outcomes are presented in Table 4. While anemia and chronic obstructive pulmonary disease (COPD) were both independently associated with both major outcome indices (30‐day readmission, and 5‐year all‐cause mortality), marital status was not associated with any of these outcomes.

**Table 4:** Multivariate logistic regression analysis for 30‐day readmission and 30‐day all‐cause mortality and multivariate Cox regression analysis for 5‐year all‐cause mortality.

	30‐day readmission		5‐year all‐cause mortality	
Index	Odds [95% CI]	*p* Value	Odds [95% CI]	*p* Value
Unmarried	0.92 [0.78–1.08]	0.29	0.98 [0.91–1.05]	0.6
Age	1 [0.99–1.01]	0.76	1.05 [1.05–1.05]	<0.001
Female	1.03 [0.87–1.21]	0.47	0.92 [0.85–0.98]	0.016
IHD	1.19 [1.02–1.38]	0.03	0.98 [0.92–1.05]	0.57
CKD	1.22 [1.04–1.43]	0.01	1.17 [1.09–1.25]	<0.001
AF	1.31 [1.12–1.52]	<0.001	1.01 [0.94–1.08]	0.75
DM	1.01 [0.87–1.18]	0.85	0.98 [0.91–1.04]	0.5
COPD	1.21 [1.01–1.47]	0.04	1.2 [1.1–1.31]	<0.001
Anemia	1.31 [1.12–1.54]	0.001	1.46 [1.36–1.57]	<0.001
PVD	1.09 [0.83–1.45]	0.53	1.3 [1.15–1.47]	<0.001

Abbreviations: AF, atrial fibrillation; CI, confidence interval; CKD, chronic kidney disease; COPD, chronic obstructive pulmonary disease; DM, diabetes mellitus; IHD, ischemic heart disease; PVD, peripheral vascular disease.

## 4 DISCUSSION

The current study, including over 7000 patients admitted for ADHF with follow‐up extending for over 5 years demonstrated that: (1) half of patients admitted for ADHF were married; (2) unmarried patients were older with a lower prevalence of traditional cardiovascular risk factors and a higher prevalence of preserved left‐ventricular function compared with married patients; (3) although numerically unmarried had poor outcomes compared with married patients, after adjustment, marital status was not independently associated with either short or long‐term outcomes.

HF emerges as one of the leading causes of morbidity and mortality worldwide associated with dire short and long outcomes, and with a trajectory for increased incidence due to the aging population. The present analysis is in line with prior reports demonstrating in‐hospital mortality of 2%–12% and 1‐year all‐cause mortality of 12%–28%.^18^, ^19^ The fact that about 70% of patients died within 5 years intensifies the need for identifying and focusing on patients at increased risk.

Beyond traditional risk factors associated with poor outcomes, psychosocial factors impact patients' outcomes as demonstrated in various cardiovascular diseases, and as acknowledged by the American Heart Association.^20^ These factors include, for example, a low socioeconomic status which was previously associated with poor outcomes and adverse events in HF patients.^3^ Marital status, as a form of social support, emerged as an independent factor associated with outcomes in patients admitted for an acute coronary syndrome.^21^, ^22^, ^23^ The association between marital status and transcatheter aortic valve implantation is more complex as married women were demonstrated to have poor long‐term outcomes while married men had a higher probability of survival.^24^ As opposed to the aforementioned associations, prior studies were not able to associate between marital status and HF outcomes. Verma et al.^7^ failed to demonstrate that having a partner for life impacts the outcomes of chronic HF. In a cohort of 357 patients admitted for ADHF, Watkins et al.^8^ demonstrated no independent association between marital status and in‐hospital death or readmission. A plausible explanation for this was provided by Wu et al.^25^ In their study of patients with HF, worse outcomes were recorded in unmarried patients, but in a multivariate analysis, after forcing medication adherence into the model, marital status was not an independent predictor of outcome. This may indicate that other indices (e.g., medication adherence, perhaps encouraged by partners), are stronger factors associated with outcome, rather than marital status.

In the present analysis, unmarried patients had nonadjusted poor outcomes compared with married patients. The fact that unmarried patients were roughly 5‐year‐older than married patients may explain why these differences were not sustained after multivariate adjustment. Further, unmarried patients had a higher prevalence of preserved LV function compared with married patients. Their admission with ADHF puts many of these patients in the HF with preserved ejection fraction (HFpEF) group, as opposed to HF with reduced ejection fraction (HFrEF) patients, and current evidence supports worse outcomes for HFpEF patients.^26^ It is also plausible that in these patients with multiple comorbidities, the impact of age, anemia, and chronic obstructive pulmonary disease is higher compared with marital status.

The lack of association between marital status and outcomes of ADHF also highlights the need for a better determinant of psychosocial indices which may impact care and hopefully outcomes. It can be postulated that marital status as a single determinant of psychosocial status is obsolete. Learning from the process of evaluating the eligibility of HF patients for a heart transplant and ventricular assist devices, it seems that combining socioeconomic status, cognitive assessment, medication adherence, and social support may be a more relevant approach to the association between psychosocial indices and outcomes.^27^

Several limitations of this study should be acknowledged. First, this was a retrospective analysis and as such, the analyses may have been skewed by unknown confounders. Second, unmarried patients represent a heterogenic group of patients including never married, widowed, and divorced. The unadjusted survival analysis demonstrated a better outcome for divorced patients compared with married and a dire outcome for widowed patients. Unfortunately, the relatively small sample size in these groups limited the ability to draw conclusions regarding this observation. Third, this study utilized data from a single center, which may limit its findings generalizability. However, the large size of the cohort, and the heterogeneity of the population in the region of this center, may somewhat mitigate this limitation.

In conclusion, marital status was not found to be independently associated with both short and long‐term outcomes of patients admitted for ADHF. Efforts for outcomes improvement should focus on more traditional risk factors such as anemia and COPD.

## CONFLICT OF INTEREST STATEMENT

The authors declare no conflict of interest.

## Data Availability

Data were generated at Shamir Medical center. Derived data supporting the findings of this study are available from the last author (S. M.) on request.
